# Insulin signaling and the regulation of insect diapause

**DOI:** 10.3389/fphys.2013.00189

**Published:** 2013-07-22

**Authors:** Cheolho Sim, David L. Denlinger

**Affiliations:** ^1^Department of Biology, Baylor UniversityWaco, TX, USA; ^2^Departments of Entomology and Evolution, Ecology, and Organismal Biology, Ohio State UniversityColumbus, OH, USA

**Keywords:** diapause, dauer, insulin signaling, FOXO, *Culex pipiens*

## Abstract

A rich chapter in the history of insect endocrinology has focused on hormonal control of diapause, especially the major roles played by juvenile hormones (JHs), ecdysteroids, and the neuropeptides that govern JH and ecdysteroid synthesis. More recently, experiments with adult diapause in *Drosophila melanogaster* and the mosquito *Culex pipiens*, and pupal diapause in the flesh fly *Sarcophaga crassipalpis* provide strong evidence that insulin signaling is also an important component of the regulatory pathway leading to the diapause phenotype. Insects produce many different insulin-like peptides (ILPs), and not all are involved in the diapause response; ILP-1 appears to be the one most closely linked to diapause in *C. pipiens*. Many steps in the pathway leading from perception of daylength (the primary environmental cue used to program diapause) to generation of the diapause phenotype remain unknown, but the role for insulin signaling in mosquito diapause appears to be upstream of JH, as evidenced by the fact that application of exogenous JH can rescue the effects of knocking down expression of ILP-1 or the Insulin Receptor. Fat accumulation, enhancement of stress tolerance, and other features of the diapause phenotype are likely linked to the insulin pathway through the action of a key transcription factor, FOXO. This review highlights many parallels for the role of insulin signaling as a regulator in insect diapause and dauer formation in the nematode *Caenorhabditis elegans.*

## Introduction

Diapause is a form of dormancy used widely by insects to survive adverse seasons. Unlike quiescence, defined as an immediate response to an unfavorable environmental stress, diapause is an anticipated, hormonally-regulated developmental arrest, frequently programmed by photoperiod. Within temperate zones, insects are temporally limited to just a few months of active development, while the remaining months are spent in diapause. Depending on the species, insect diapause can occur in embryos (e.g., the commercial silkmoth *Bombyx mori*), larvae (e.g., southwestern corn borer *Diatraea grandiosella*), pupae (e.g., flesh fly *Sarcophaga crassipalpis*) or adults (e.g., mosquito *Culex pipiens*). Among species in temperate zones, an overwintering diapause is most common, but a summer diapause can also occur (Masaki, 1980), and diapause is also well-documented among tropical species (Denlinger, 1986). In temperate zones the shortening day lengths and declining temperatures of late summer and early autumn provide the dominant environmental cues signaling the advent of winter (Tauber et al., 1986; Kostal and Denlinger, 2011), cues that set into motion a series of preparatory steps for successful overwintering.

The environmental cues used to program diapause are frequently received long before the actual inception of diapause. Depending on the species, the photoperiodic signals are received either through the eyes or directly by light-sensitive cells within the brain (Goto et al., 2010; Numata and Udaka, 2010). Most evidence suggests that the circadian clock is involved in distinguishing short from long days (Saunders, 2012; Goto, 2013; Meuti and Denlinger, 2013). The transduction pathway for photoperiodic stimuli engages neurons in the *pars intercerebralis*, *pars lateralis* and other domains within the brain (Shiga and Numata, 2000; Shimokawa et al., 2008) that release neuropeptides or growth factors into neighboring or remote cells to regulate development. Among the targets of these neuropeptides are endocrine glands such as the *corpora cardiaca*, the *corpora allata* and the prothoracic gland, organs that in turn synthesize and release hormones including juvenile hormones, ecdysteroids, adipokinetic hormone, as well as additional neuropeptides that impact insect diapause.

A functional module approach is a helpful way to view the diapause mechanism (Emerson et al., 2009; Bradshaw and Holzapfel, 2010). Three candidate modules are proposed: an input module that includes the functional timekeeping mechanism, an intermediate module linking photoperiodism to hormonal events, and an output module that includes the physiological responses. Modularity of this sort has been commonly invoked to interpret genetic mechanisms of embryonic development such as pattern formation and differentiation (Raff, 1996). Key components in a module are signaling cascades such as hedgehog, transforming growth factor (TGF-β) and insulin signaling (Cohen, 2003; Dupont and Holzenberger, 2003; Logan and Nusse, 2004; Bray, 2006; Kitisin et al., 2007). If we consider diapause as an alternative developmental program with separate functional modules, the application of this concept may be useful for dissecting molecular mechanisms of diapause programs.

**Figure 1:**
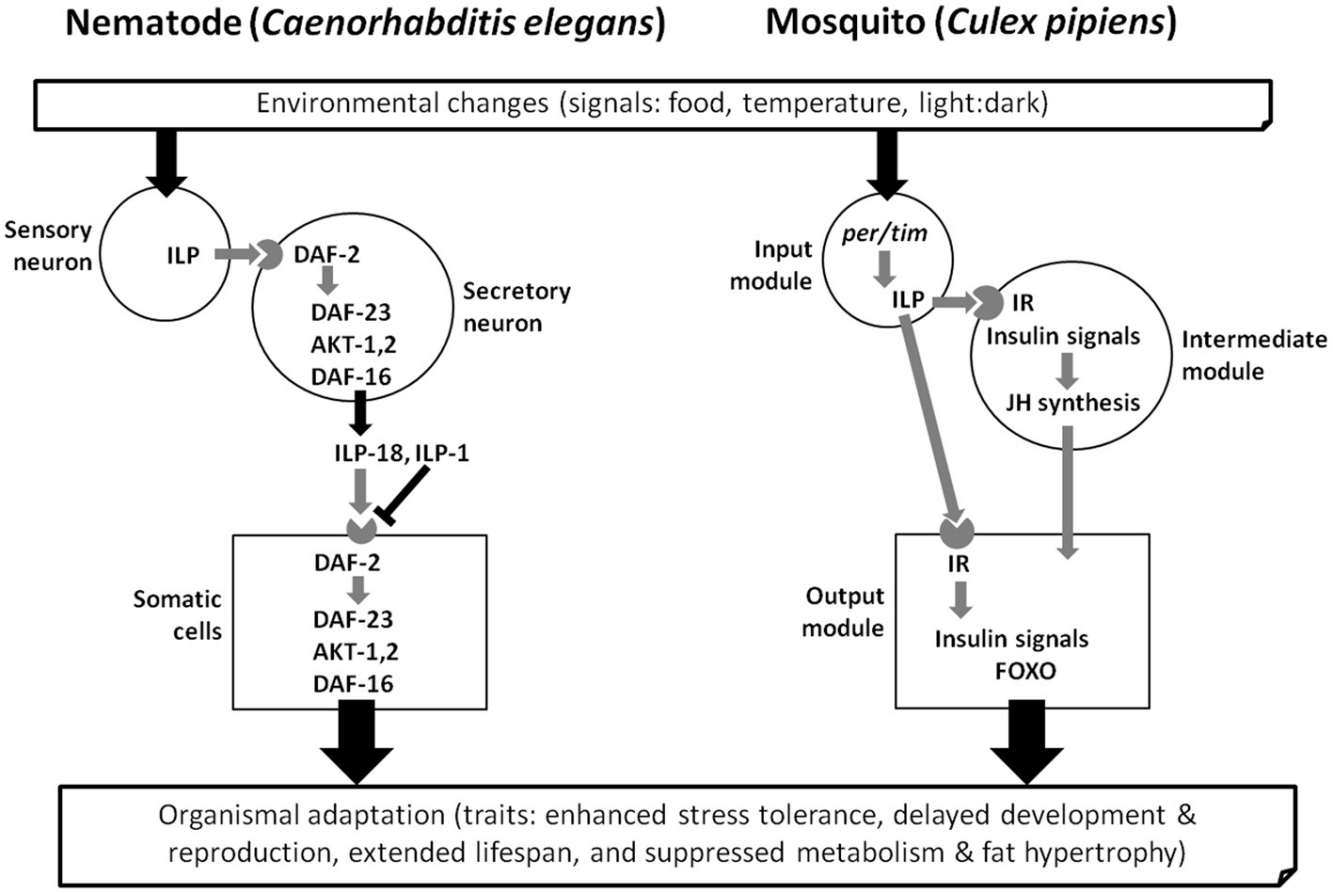
**Three conceptual modules (input, intermediate and output) that influence dauer larva formation in the nematode *Caenorhabditis elegans* and adult diapause of the mosquito *Culex pipiens*, based on references in the text.** Gene activation (Black) and deactivation (Gray). Insulin-like peptide, ILP; DAF-2, insulin receptor (IR); DAF-23, phosphoinoitide 3-kinase; Akt, protein kinase B; DAF-16, forkhead transcription factor (FOXO); *per*/*tim*, core genes of molecular clock system; JH, juvenile hormone. The mosquito FOXO and insulin receptor (IR) are homologs of nematode DAF-16 and DAF-2, respectively.

Several recent reviews discuss regulatory features of diapause such as molecular regulation (Denlinger, 2002; Robich and Denlinger, 2005; Macrae, 2010; Williams et al., 2010), hormonal control (Denlinger et al., 2012), the circadian clock and photoperiodism (Goto et al., 2010; Saunders, 2010; Kostal, 2011), and energy utilization (Hahn and Denlinger, 2007, 2011). One unifying theme for diapause in diverse species may be insulin signaling (Tatar and Yin, 2001; Williams et al., 2006; Sim and Denlinger, 2008, 2009a). This signaling pathway has been linked to diverse features of the diapause phenotype including arrested reproduction, extended lifespan, suppressed metabolism, fat hypertrophy and enhanced stress tolerance. Dauer formation in the nematode *Caenorhabditis elegans* (Figure 1) offers many parallels to insect diapause, including a role for insulin signaling, thus the comprehensive understanding of the molecular basis for dauer formation (Gottlieb and Ruvkun, 1994; Kimura et al., 1997; Apfeld and Kenyon, 1998) provides valuable insights for exploring general patterns of developmental arrest in invertebrate systems. The goal of this review is to summarize evidence linking insulin signaling to insect diapause and to thus create a foundation for developing a comprehensive view of the role of this pathway in shaping the complex diapause phenotype.

## Components of the insulin signaling pathway

Insulin signaling has been implicated as a regulator of diapause by observing the effects of this pathway on developmental and metabolic suppression (Apfeld and Kenyon, 1998; Tatar et al., 2001; Hahn and Denlinger, 2007; Sim and Denlinger, 2008; Ragland et al., 2010; Williams et al., 2010) and by observing naturally segregating variation of PI3K, a member of the insulin signaling pathway, in association with adult reproductive diapause in *Drosophila melanogaster* (Williams et al., 2006).

Seven genes encode insulin-like peptides in *Drosophila* (Brogiolo et al., 2001). All of these peptides have a domain structure that produces two chains, resulting in active dimer formation (Leevers, 2001). *Drosophila* insulin receptor (receptor tyrosine kinases) shares sequence similarity with human insulin receptor and can be activated by insulin (Fernandez et al., 1995; Chen et al., 1996). Activated receptor tyrosine kinases (RTKs) activate phosphatidylinositol 3-kinase (PI3K) through direct binding or through tyrosine phosphorylation of scaffolding adaptors, such as IRS1, which then bind and activate PI3K. PI3K phosphorylates phosphatidylinositol-4,5-bisphosphate (PIP_2_) to generate phosphatidylinositol-3,4,5-trisphosphate (PIP_3_) (Britton et al., 2002). In the end, PI3K targets two intracellular signaling proteins, Akt (also known as Protein Kinase B, PKB) and 3-phophoinositide-dependent protein kinase-1 (PDK-1); phosphorylation at serine and threonine residues activates Akt and PDK-1 (Taniguchi et al., 2006).

Akt plays a key role in multiple cellular processes including glucose metabolism, apoptosis, cell proliferation, transcription and cell migration (Hanada et al., 2004; Fayard et al., 2005). These various cellular processes are mediated by transcriptional factors and kinases which are activated by Akt phosphorylation. The downstream molecules of Akt include forkhead of transcriptional factors (FOXs), glycogen synthase kinase-3 (GSK-3), tuberous sclerosis complex (TSC1/2) and Rab-GTPase-activating protein (Rab-GAP) (Frame et al., 2001; Harris and Lawrence, 2003; Junger et al., 2003; Sano et al., 2003). In addition, a number of upstream proteins regulate Akt; these include protein phosphatase 2A (PP2A), wideborst (Wdb), and a PH-domain leucine-rich repeat protein phosphatase (PHLPP) (Du et al., 2003; Vereshchagina et al., 2008).

Several proteins are involved in regulation of the insulin receptor and thus influence intracellular signaling components in the insulin signaling pathway. Protein Tyrosine Phosphatase 1B dephosphorylates active insulin receptor (Elchebly et al., 1999), while suppressors of cytokine signaling (SOCS)-1 and SOCS-3 can bind insulin receptor substrate, and attenuate insulin signals (Ueki et al., 2004). Other molecules including extracellular-signal-regulated kinase (ERK), Jun-N-terminal kinases, and kinase S6 attenuate the activity of insulin receptor (Miller et al., 1996; Bouzakri et al., 2003; Harrington et al., 2004). Phosphatase and tensin homolog (PTEN) can also inactivate PIP_3_, a midpoint in the insulin signaling pathway (Goberdhan et al., 1999). Comprehensive reviews describe details of structure, signaling, function of insulin-like peptides, as well as other components of the insulin signaling pathway (Luckhart and Riehle, 2007; Antonova et al., 2012).

One outstanding question that remains is how input from the photoperiodic clock is linked to the insulin signaling pathway. Several candidate neuropeptides could serve as intermediaries. For example, Pigment Dispersing Factor (PDF) appears to be an output molecule of the circadian clock system in several insects and has been extensively studied in *D. melangaster* (Meelkop et al., 2011). Additional neuropeptides in flies, short neuropeptide F (sNPF), corazonin (CRZ) and drosulfakinins (DSKs), function in regulating insulin production, and thus could also serve an intermediary role. Some insulin-producing cells (IPCs) in the fly brain also coexpress receptors of both sNPF and CRZ neuropeptides, as well as the ligand for DSKs (Kapan et al., 2012; Soderberg et al., 2012). In these studies, knockdown of either sNPF or DSK decreases transcription of ILPs in the brain, suggesting a regulatory action of these two neuropeptides on IPCs. In *Rhodnius prolixus*, levels of prothoracicotrophic hormone (PTTH) oscillate in a circadian manner, suggesting a possible link between PTTH and clock functions as well (Vafopoulou et al., 2007, 2012). Yet, links between these neuropeptides, insulin signaling, and insect diapause remain to be determined.

## Cell cycle and developmental regulation

Arrest of the cell cycle and development are key characteristics of diapause. Pupal diapause in the flesh fly involves a G0/G1 cell cycle arrest that appears to be controlled by down regulation of *proliferating cell nuclear antigen* (Tammariello and Denlinger, 1998), a gene that controls the cell cycle by direct interaction with the cyclin/cdk complex (Watanabe et al., 1998). FOXO proteins have known roles in inducing cell cycle arrest. For example, in dauer larvae of *Caenorhabditis elegans* a FOXO homolog induces a G0/G1 cell cycle arrest through induction of Cip/Kip inhibitor, Cki-1 (Boxem and Van Den Heuvel, 2001). A similar role for FOXO is evident in *Drosophila* (Kramer et al., 2003), in which activated dFOXO promotes a G1 cell cycle arrest.

*Drosophila* females reared under low temperature and short daylength enter an adult reproductive diapause characterized by arrest of ovarian development in the previtellogenic stage, while non-diapausing females initiate vitellogenesis and complete ovarian development (Tatar et al., 2001). Development of *Drosophila* ovaries is regulated by an insulin signal in germ cells; dILPs specifically control the G2 phase of germ cell cycle via PI3K and dFOXO (LeFever and Drummond-Barbosa, 2005; Hsu et al., 2008). Regulation of ovarian development by insulin signaling is not limited to *Drosophila* but is also evident in the mosquito *Culex pipiens*. The insulin signal/FOXO pathway is central to initiation of the diapause program, including ovarian development arrest. A “diapause-like” ovarian arrest can be simulated in non-diapausing females by knocking down the insulin receptor (InR) using RNAi; this knock-down effect can be reversed with application of juvenile hormone, an endocrine stimulant well-known to terminate diapause in this species (Sim and Denlinger, 2008). Insulin-like peptide 1 (ILP-1) is the ILP most likely implicated in the diapause response of *Cx. pipiens* (Sim and Denlinger, 2009a).

In *C. elegans*, low food levels prompt synthesis of high levels of dauer pheromone, which in turn lead to dauer formation, rather than reproductive growth (Golden and Riddle, 1982, 1984). These environmental cues initially alter insulin signaling by regulating insulin-like peptide synthesis and secretion in specific subsets of sensory neurons. *C. elegans* has only a single insulin receptor (daf-2), but it has genes encoding 40 putative insulin-like peptides (Flatt et al., 2008a). Daf-2 is implicated in many genetically separable processes, including the dauer decision, lifespan control, and reproductive timing (Flatt et al., 2008b; Lee et al., 2008). Mutants with reduced daf-2 activity enter the dauer state, while increased insulin signaling promotes germ line proliferation, resulting in an increase of germ line stem cells. Many of the ILPs are expressed in sensory neurons and interneurons, where they encode distinct environmental information to regulate initiation and termination of dauer formation. For example, ILP-1(ins-1) induces dauer arrest under low food levels, and under favorable food conditions, daf-28 (insulin-like peptide) inhibits dauer arrest, whereas ILP-6 (ins-6) promotes the transition from the dauer state to normal reproductive growth (Cornils et al., 2011).

The finding that ILPs in *C. elegans* encode environmental cues used to regulate physiology also reflects what is found in insects, such as the fly *D. melanogaster* and the mosquito *C. pipiens*. *D. melanogaster* has genes encoding 7 ILPs, and as in *C. elegans*, the ILPs are expressed in different sensory neurons and interneurons. Interestingly, some of these neuronally-expressed ILPs (dilp-2, -3, -5) have been proposed to regulate growth and metabolism (Ikeya et al., 2002; Broughton et al., 2008; Zhang et al., 2009). We thus argue that insulin signals are likely used as mediators of a wide range of environmental cues, including those involved in regulating diverse forms of developmental arrest (Figure 1).

## Lifespan extension

Genetic studies using the nematode *C. elegans* and the fruit fly *D. melanogaster* have identified several genes involved in extending lifespan. Work on the dauer stage of *C. elegans* is at the forefront of such research. Genome-wide RNAi screens identified key functional groups in the *C. elegans* insulin signaling pathway that contribute to lifespan extension (Hamilton et al., 2005). Most mutants with reduced insulin-like signaling have both dauer and extended lifespan responses, but in some cases there are distinct differences when and where insulin/FOXO signals are activated. Most studies on daf-2 suggest it functions within the nervous system to regulate lifespan as well as dauer arrest (Kimura et al., 1997; Apfeld and Kenyon, 1998). Yet, several studies on the target of insulin signaling, daf-16 (aka forkhead of transcriptional factor FOXO), suggest that dauer arrest and lifespan are regulated by FOXO activity in a different way: FOXO within the nervous system has a stronger influence on dauer arrest than on lifespan, whereas intestinal FOXO plays a greater role in regulating lifespan than in regulating dauer arrest (Libina et al., 2003). Thus, FOXO activation in different tissues may have distinct phenotypic consequences. Furthermore, insulin signaling during larval development regulates dauer arrest without significantly impacting lifespan, whereas insulin-like signaling during adulthood regulates lifespan (Dillin et al., 2002). Daf-2 is thought to activate a conserved PI-3 kinase signaling pathway that affects lifespan, at least in part by regulating nuclear localization of daf-16 (FOXO). This transcription factor, FOXO, appears to extend lifespan by activating its downstream genes products such as superoxide dismutase, metallothionin, catalase, glutathione S-transferase, small heat shock proteins, and apolipoprotein (Vanfleteren and De Vreese, 1995; Honda and Honda, 1999; Barsyte et al., 2001; Sun et al., 2002; Walker and Lithgow, 2003).

Diapause incidence in *D. melanogaster* varies among populations (Schmidt et al., 2005; Williams et al., 2006). A transgenic study of Dp110 (phosphoinositide 3-kinase), a member of the insulin signaling pathway, supports the view that this gene, and hence the insulin signaling pathway, plays an important role in induction of reproductive diapause (Williams et al., 2006). Knock-down of genes encoding insulin-like peptides, insulin receptor and CHICO (IRS), and overexpression of the downstream transcription factor dFOXO, as well as inhibitor studies using the PIP_3_ inhibitor PTEN, all reduce insulin signaling and subsequently extend lifespan (Clancy et al., 2001; Giannakou et al., 2004; Hwangbo et al., 2004; Giannakou and Partridge, 2007; Lee et al., 2008; Demontis and Perrimon, 2010; Gronke et al., 2010). Likewise, induction of the tuberous sclerosis complex (TSC1/2), kinase S6, the dFOXO regulated histone deacetylase Sir2, and the insulin signal suppressing pathway Jun-N-terminal kinase (JNK) extend lifespan (Kapahi et al., 2004; Partridge et al., 2005; Wang et al., 2005).

Furthermore, insulin signals appear to be a regulator of juvenile hormone synthesis (Flatt et al., 2005; Tu et al., 2005). In *Drosophila*, insulin receptors are present in the *corpora allata* (CA), the glands that synthesize JH (Belgacem and Martin, 2006), and suppression of the insulin signal correlates with low JH production (Tatar and Yin, 2001; Tu et al., 2005). Knock-down of the insulin receptor in the CA concurrently suppresses the gene encoding 3-hydroxy-3-methylglutargyl CoA Reductase (HMGCR), a key enzyme in JH synthesis (Belgacem and Martin, 2007). The subsequent shut-down of JH synthesis is a key signaling event triggering the onset of adult reproductive diapause in many insects (Denlinger et al., 2005), including *D. melanogaster*, the mosquito *Cx. pipiens* and the butterfly *Danaus plexippus* (Herman, 1981; Herman and Tatar, 2001; Sim and Denlinger, 2008). *D. melanogaster* selected to survive a high dose of JH analog overcame lifespan reduction when compared to flies not receiving JH (Flatt and Kawecki, 2007). These lines of evidence suggest that JH is involved in the trade-off between reproduction and extended lifespan through the insulin signaling pathway.

## Suppressed metabolism and fat hypertrophy

At the initiation of diapause, metabolic processes are coordinately downregulated, thus enabling the overwintering insect to economically utilize its energy reserves, but in addition, this metabolic downregulation helps to minimize deficiencies in cellular processes that can cause cell death (Hand et al., 2011). Diapausing insects remain hypometabolic even after temperatures revert to conditions favorable for development. However, certain metabolic genes involved in the accumulation of energy reserves are highly upregulated, especially during the preparatory period of diapause. This energy storage is critical not only for surviving prolonged periods of developmental arrest but also for maximizing reproductive success once development resumes (Hahn and Denlinger, 2007). Carbohydrate sources such as nectar and rotten fruit are critical carbohydrate sources used for increasing energy reserves in adult diapausing females of the mosquito *Cx. pipiens*, the butterfly *Danaus plexippus*, and some other diapausing insects (Alonsomejia et al., 1997; Robich and Denlinger, 2005; Reynolds et al., 2012). We generated transcript profiles of thirty-two fat-related genes during diapause in the mosquito *Cx. pipiens* (Sim and Denlinger, 2009b), and among the genes up-regulated in early diapause were *fatty acid synthase-1, -3*, and *fatty acid binding protein*, genes that contribute to accumulation of triacylglycerides in the fat body. This result is consistent with the observation that this mosquito switches from blood feeding to sugar feeding as a component of the diapause program and more than doubles its lipid reserves compared with females programmed for continuous development. When we knocked down *foxo* transcript by injection of dsRNA into these diapausing mosquitoes, we observed an immediate halt in the accumulation of lipid reserves (Sim and Denlinger, 2008). FOXO is normally activated by suppression of insulin signaling; thus FOXO may be involved in increasing transcript levels of genes involved in fatty acid synthesis, as observed in newly-emerged diapausing females. A transcriptome analysis of the Asian tiger mosquito, *Aedes albopictus,* also suggests the importance of FOXO during early diapause (Poelchau et al., 2011).

Insulin signaling and its target FOXO are implicated as a major regulator of diapause through effects on metabolic suppression, fat hypertrophy, and growth control (Puig et al., 2003; Williams et al., 2006; Hahn and Denlinger, 2007; Sim and Denlinger, 2009a, b; Ragland et al., 2010). A transcriptome analysis of diapause termination in the apple maggot fly, *Rhagoletis pomonella*, reveals the importance of the TOR signaling pathway, a pathway that interacts with insulin signaling (Ragland et al., 2011). TSC1 and TSC2, negative regulators of TOR signals and regulators of cell growth, were significantly upregulated in late diapause. FOXO and TOR pathways are both linked to insulin signaling and offer links for integrating metabolic and growth responses.

Among the *Drosophila* insulin-like peptides, ligands 1-5 are predicted to be closely related to mammalian insulin, while ligands 6 and 7 are more similar to IGF-1 and relaxin, respectively (Brogiolo et al., 2001). Four of these insulin-like peptides, 1, 2, 3, and 5 are expressed in insulin-producing cells (IPCs) in the brain (Cao and Brown, 2001; Ikeya et al., 2002; Rulifson et al., 2002), and loss of insulin-like peptide-producing cells or mutations in the gene for *Drosophila* insulin receptor (dInR) or CHICO results in a significant increase in triacylglycerides (Bohni et al., 1999; Tatar et al., 2001; Broughton et al., 2005). By contrast, insulin is a positive regulator of fat cell mass, acting through changes in both cell number and lipid storage (Diangelo and Birnbaum, 2009). This evidence suggests that, unlike in mammals, different insect ILP may be involved in regulating distinct physiological processes such as energy metabolism, fat cell proliferation, lipid storage and other key traits for survival. Thus, it will be important to know when and where particular insulin-like peptides are suppressed or activated, and how the insulin-like peptides generate the increased fat cell mass and lipid storage in diapausing insects.

## Enhanced stress tolerance

Diapausing insects are particularly well-adapted to survive low temperatures and other forms of environmental stress (Denlinger and Lee, 2010). Cold hardiness is frequently a component of the diapause program but is sometimes acquired after the onset of diapause, in direct response to low temperature (Denlinger, 1991). A variety of molecular mechanisms are used to either avoid or survive freezing (Michaud and Denlinger, 2006, 2007; Khani and Moharramipour, 2010; Vesala and Hoikkala, 2011). Suppression of the insulin signal appears to induce physiological responses promoting resistance to low temperature, oxidative stress, and pathogenic infections (Clancy et al., 2001; Broughton et al., 2005; Zhang et al., 2009; Felix et al., 2012). In *C. elegans*, dauer larvae differ from non-dauer larvae in aspects of metabolism related to cold tolerance. Genes involved in trehalose synthesis are upregulated in daf-2 (insulin receptor) mutants and dauers (Wang and Kim, 2003; McElwee et al., 2006; Shmookler Reis et al., 2011). Fatty acid desaturase genes are essential for cold tolerance in many animals, an effect promoted by the preservation of membrane fluidity at sub-zero temperatures (Gracey et al., 2004; Brock et al., 2007; Murray et al., 2007). Cold tolerance in dauers is enhanced by the overlapping effect of genes encoding fatty acid desaturase, targeted by insulin signal/FOXO, and genes involved in the cold-induced stress response (Savory et al., 2011).

*Drosophila* FOXO has a critical role in the systemic regulation of antioxidant enzymes, a response that acts through the insulin/FOXO signaling pathway in insulin-producing cells (IPCs) (Kops et al., 2002b; Hwangbo et al., 2004). FOXO activation subsequently increases stress tolerance through up-regulation of superoxide dismutases (Kops et al., 2002a). This genetic regulation of antioxidant enzymes by FOXO is also noted in *C. elegans* (Vanfleteren and De Vreese, 1995) and the mosquito *Cx. pipiens* (Sim and Denlinger, 2011). Dauer worms and diapausing mosquitoes increase expression of the protective enzymes superoxide dismutase and catalase. In addition, several genome-wide studies indicate that detoxification/stress response genes are among the most common group of genes regulated by the insulin/FOXO signaling pathway (Murphy et al., 2003; Oh et al., 2006; Gershman et al., 2007).

The insulin signaling pathway also plays a critical role in regulation of innate immunity and lifespan in many insects (Luckhart and Riehle, 2007). However, there is a dichotomy in the functional role of insulin signals in activation of the immune response. For example, in the mosquito *Anopheles stephensi*, increased Akt/PKB signaling in the midgut significantly reduces malaria parasite development compared to control mosquitoes (Corby-Harris et al., 2010). The brain of the mosquito *Aedes aegypti* releases insulin-like peptides (ILPs) in response to a blood meal. In turn, the insulin signal induces hemocyte (immune cells) production, which serve as the first line of defense against pathogenic infections (Castillo et al., 2011). By contrast, in *C. elegans*, insulin signals are linked to both innate immunity and extended lifespan. The loss of function of insulin receptor (daf-2) results in decreased insulin signaling and enhanced resistance to pathogenic bacterial infection (Garsin et al., 2003). When forkhead transcription factor (daf-16), which is negatively regulated by the insulin signaling pathway in *C. elegans*, is suppressed the worms exhibit increased susceptibility to infection by pathogenic bacteria. Similar results were found in the fly *D. melanogaster*, in which there is a link between the Toll signaling pathway, the pathway that activates the innate immune response, and the insulin signaling pathway (Diangelo et al., 2009). These lines of evidence suggest that insulin/FOXO signaling in diapausing insects may be linked to induction of immune effectors that enhance resistance to pathogenic infection.

## Future direction

The evidence we present links the insulin/FOXO signaling pathway to insect diapause characteristics including cell cycle arrest, developmental arrest, extended lifespan, suppressed metabolism, fat hypertrophy, and enhanced stress tolerance. Although relatively few insect species have been examined, involvement of this pathway may emerge as one of the unifying themes of insect diapause. In life-history studies, the insulin/FOXO signaling pathway appears to also regulate growth, reproduction, and lifespan in numerous species including flies, worms and mosquitoes. Phenotypic plasticity observed in life-history traits provides insights for understanding relationships among diapause characteristics. Similar phenotypic plasticity of fitness factors is evident in diapause, i.e., trade-offs between/among diapause characteristics. For example, in adult reproductive diapause, extended lifespan is frequently coupled with arrested ovarian development and suppressed metabolic rate. Since insulin signaling plays a role in phenotypic plasticity among fitness factors, we propose that the insulin signal is a key regulator among diapause characteristics including suppressed growth and metabolism, enhanced stress tolerance, and extended lifespan. This idea raises several new questions. First, what is the nature and extent of the linkages among modules through insulin signals? Second, how are changes in insulin signals within one module coordinated with others, and what are the mechanisms that promote systemic changes? The answers to these questions are not simple, but recent advances in genomics and functional genetics provide new opportunities for testing hypotheses of this nature. Hopefully, such experiments will enable us to pinpoint the molecular mechanisms of phenotypic plasticity associated with insect diapause.

Several lines of evidence support our proposition. First, recent studies found that insulin signals can act locally as well as systemically in the fruit fly *D. melanogaster*. The glia provide local signals necessary for activation of neighboring neuroblasts, and interestingly the glia also produce insulin-like peptides (ILPs) that respond to signals from the fat body by binding to receptors on larval neuroblasts (Chell and Brand, 2010; Sousa-Nunes et al., 2011). Considering the fact that the brain, endocrine organs (*corpora cardiac, corpora allata*, prothoracic gland), and fat body are all key organs essential to the diapause response, the presence of a systemic signaling system operating among these organs is likely to offer a conduit for cross-talk that may be critical for implementing and coordinating a successful diapause program (Xu et al., 2012). With the insulin signaling pathway being involved in so many aspects of the diapause phenotype, the local and systemic signals from different insulin-like peptides are promising candidates to explain molecular mechanisms used to generate this phenotype.

Secondly, modularity is a suitable model for viewing the complicated molecular mechanisms of diapause. Circadian clock oscillations (input module) are certainly functioning in the insect brain and likely contribute to photoperiodism (Ito et al., 2008; Ikeno et al., 2010). Interestingly, the clock genes have linkages to insulin signaling (Allen, 2007; Zheng and Sehgal, 2010). Diapause incidence in *Drosophila* is elevated when PI3-kinase, an insulin-regulated gene, is upregulated and is lowered when this gene is downregulated (Williams et al., 2006). This connection most likely acts through Susi, an inhibitor of insulin-regulated PI3-kinase. Susi shows a circadian pattern of expression that is high at night and low during the day (Claridge-Chang et al., 2001; McDonald et al., 2001; Wittwer et al., 2005). We suggest that insulin signaling is suppressed by long nightlengths (short daylengths), which in turn suppresses juvenile hormone synthesis within the *corpora allata* (intermediate module) (Hardie et al., 1985; Tatar et al., 2001; Tu et al., 2005). The fat body (output module) is crucial to important physiological functions including nutrient sensing, lipid storage, and endocrine signaling to the brain and reproductive organs. Additionally, the fat body is the nexus for lipid storage, arrested reproductive development, and induced stress tolerance during diapause. Insulin/FOXO appears to coordinate, or at least be involved in, the physiological responses during each module of adult reproductive diapause (Sim and Denlinger, 2008, 2009a, 2011, 2013). However, we still lack details and insight into how insulin signals affect each module of diapause at both local and systemic levels and to what extent insulin/Foxo signals are involved in diapauses of different developmental stages. Diapause appears to have evolved multiple times in insect lineages, thus we can very well-expect species variation in how this signaling pathway is exploited for regulating diapause in different species. The exciting prospect is that the pervasive influence of insulin signaling offers connections to insect diapause at many levels, from connections to photoperiodism through to the downstream generation of many of the phenotypic characteristics of the diapause state.

### Conflict of interest statement

The authors declare that the research was conducted in the absence of any commercial or financial relationships that could be construed as a potential conflict of interest.

## References

[B1] Allen M. J. (2007). What makes a fly enter diapause. Fly (Austin) 1, 307–310 18820432 10.4161/fly.5532

[B2] Alonsomejia A. Rendonsalinas E. Montesinospatino E. Brower L. P. (1997). Use of lipid reserves by monarch butterflies overwintering in Mexico: implications for conservation. Ecol. Appl. 7, 934–947 10.1890/1051-0761(1997)007[0934:UOLRBM]2.0.CO;2

[B3] Antonova Y. Arik A. J. Moore W. Riehle M. A. Brown M. R. (2012). Insulin-like peptides: structure, signaling, and function, in Insect Endocrinology, ed Gilbert L. I. (San Diego, CA: Academic Press), 63–92

[B4] Apfeld J. Kenyon C. (1998). Cell nonautonomy o. *C. elegans* daf-2 function in the regulation of diapause and life span. Cell 95, 199–210 10.1016/S0092-8674(00)81751-1 9790527

[B5] Barsyte D. Lovejoy D. A. Lithgow G. J. (2001). Longevity and heavy metal resistance in daf-2 and age-1 long-lived mutants of *Caenorhabditis elegans*. FASEB J. 15, 627–634 10.1096/fj.99-0966com 11259381

[B6] Belgacem Y. H. Martin J. R. (2006). Disruption of insulin pathways alters trehalose level and abolishes sexual dimorphism in locomotor activity in *Drosophila*. J. Neurobiol. 66, 19–32 10.1002/neu.20193 16187303

[B7] Belgacem Y. H. Martin J. R. (2007). Hmgcr in the *corpus allatum* controls sexual dimorphism of locomotor activity and body size via the insulin pathway in *Drosophila*. PLoS ONE 2:e187 10.1371/journal.pone.0000187 17264888 PMC1779623

[B8] Bohni R. Riesgo-Escovar J. Oldham S. Brogiolo W. Stocker H. Andruss B. F. (1999). Autonomous control of cell and organ size by CHICO, a *Drosophila* homolog of vertebrate IRS1-4. Cell 97, 865–875 10.1016/S0092-8674(00)80799-0 10399915

[B9] Bouzakri K. Roques M. Gual P. Espinosa S. Guebre-Egziabher F. Riou J. P. (2003). Reduced activation of phosphatidylinositol-3 kinase and increased serine 636 phosphorylation of insulin receptor substrate-1 in primary culture of skeletal muscle cells from patients with type 2 diabetes. Diabetes 52, 1319–1325 10.2337/diabetes.52.6.1319 12765939

[B10] Boxem M. Van Den Heuvel S. (2001). lin-35 Rb and cki-1 Cip/Kip cooperate in developmental regulation of G1 progression in *C. elegans*. Development 128, 4349–4359 11684669 10.1242/dev.128.21.4349

[B11] Bradshaw W. E. Holzapfel C. M. (2010). Light, time, and the physiology of biotic response to rapid climate change in animals. Annu. Rev. Physiol. 72, 147–166 10.1146/annurev-physiol-021909-135837 20148671

[B12] Bray S. J. (2006). Notch signalling: a simple pathway becomes complex. Nat. Rev. Mol. Cell Biol. 7, 678–689 10.1038/nrm2009 16921404

[B13] Britton J. S. Lockwood W. K. Li L. Cohen S. M. Edgar B. A. (2002). *Drosophila*'s insulin/PI3-kinase pathway coordinates cellular metabolism with nutritional conditions. Dev. Cell 2, 239–249 10.1016/S1534-5807(02)00117-X 11832249

[B14] Brock T. J. Browse J. Watts J. L. (2007). Fatty acid desaturation and the regulation of adiposity in *Caenorhabditis elegans*. Genetics 176, 865–875 10.1534/genetics.107.071860 17435249 PMC1894614

[B15] Brogiolo W. Stocker H. Ikeya T. Rintelen F. Fernandez R. Hafen E. (2001). An evolutionarily conserved function of the *Drosophila* insulin receptor and insulin-like peptides in growth control. Curr. Biol. 11, 213–221 10.1016/S0960-9822(01)00068-9 11250149

[B16] Broughton S. Alic N. Slack C. Bass T. Ikeya T. Vinti G. (2008). Reduction of DILP2 in *Drosophila* triages a metabolic phenotype from lifespan revealing redundancy and compensation among DILPs. PLoS ONE 3:e3721 10.1371/journal.pone.0003721 19005568 PMC2579582

[B17] Broughton S. J. Piper M. D. Ikeya T. Bass T. M. Jacobson J. Driege Y. (2005). Longer lifespan, altered metabolism, and stress resistance in *Drosophila* from ablation of cells making insulin-like ligands. Proc. Natl. Acad. Sci. U.S.A. 102, 3105–3110 10.1073/pnas.0405775102 15708981 PMC549445

[B18] Cao C. Brown M. R. (2001). Localization of an insulin-like peptide in brains of two flies. Cell Tissue Res. 304, 317–321 10.1007/s004410100367 11396725

[B19] Castillo J. Brown M. R. Strand M. R. (2011). Blood feeding and insulin-like peptide 3 stimulate proliferation of hemocytes in the mosquito *Aedes aegypti*. PLoS Pathog. 7:e1002274 10.1371/journal.ppat.1002274 21998579 PMC3188524

[B20] Chell J. M. Brand A. H. (2010). Nutrition-responsive glia control exit of neural stem cells from quiescence. Cell 143, 1161–1173 10.1016/j.cell.2010.12.007 21183078 PMC3087489

[B21] Chen C. Jack J. Garofalo R. S. (1996). The *Drosophila* insulin receptor is required for normal growth. Endocrinology 137, 846–856 10.1210/en.137.3.846 8603594

[B22] Clancy D. J. Gems D. Harshman L. G. Oldham S. Stocker H. Hafen E. (2001). Extension of life-span by loss of CHICO, a *Drosophila* insulin receptor substrate protein. Science 292, 104–106 10.1126/science.1057991 11292874

[B23] Claridge-Chang A. Wijnen H. Naef F. Boothroyd C. Rajewsky N. Young M. W. (2001). Circadian regulation of gene expression systems in the *Drosophila* head. Neuron 32, 657–671 10.1016/S0896-6273(01)00515-3 11719206

[B24] Cohen M. M. Jr. (2003). The hedgehog signaling network. Am. J. Med. Genet. A 123A 5–28 10.1002/ajmg.a.20495 14556242

[B25] Corby-Harris V. Drexler A. Watkins De Jong L. Antonova Y. Pakpour N. Ziegler R. (2010). Activation of Akt signaling reduces the prevalence and intensity of malaria parasite infection and lifespan in *Anopheles stephensi* mosquitoes. PLoS Pathog. 6:e1001003 10.1371/annotation/738ac91f-8c41-4bf5-9a39-bddf0b777a89 20664791 PMC2904800

[B26] Cornils A. Gloeck M. Chen Z. Zhang Y. Alcedo J. (2011). Specific insulin-like peptides encode sensory information to regulate distinct developmental processes. Development 138, 1183–1193 10.1242/dev.060905 21343369 PMC3042873

[B27] Demontis F. Perrimon N. (2010). FOXO/4E-BP signaling in *Drosophila* muscles regulates organism-wide proteostasis during aging. Cell 143, 813–825 10.1016/j.cell.2010.10.007 21111239 PMC3066043

[B28] Denlinger D. L. (1986). Dormancy in tropical insects. Annu. Rev. Entomol. 31, 239–264 10.1146/annurev.en.31.010186.001323 3510585

[B29] Denlinger D. L. (1991). Relationship between cold-hardiness and diapause, in Insects at Low Temperature, eds Lee R. E. Denlinger D. L. (New York, NY: Chapman and Hall), 174–198

[B30] Denlinger D. L. (2002). Regulation of diapause. Annu. Rev. Entomol. 47, 93–122 10.1146/annurev.ento.47.091201.145137 11729070

[B31] Denlinger D. L. Lee R. E. (2010). Low Temperature Biology of Insects, Cambridge: Cambridge University Press 10.1017/CBO9780511675997

[B32] Denlinger D. L. Yocum G. D. Rinehart J. P. (2005). Hormonal control of diapause, in Comprehensive Molecular Insect Science, eds Gilbert L. I. Iatrou K. Gill S. S. (Amsterdam: Elsevier), 615–650

[B33] Denlinger D. L. Yocum G. D. Rinehart J. P. (2012). Hormonal control of diapause, in Insect Endocrinology, ed Gilbert L. I. (Amsterdam: Elsevier), 430–463

[B34] Diangelo J. R. Birnbaum M. J. (2009). Regulation of fat cell mass by insulin in *Drosophila melanogaster*. Mol. Cell. Biol. 29, 6341–6352 10.1128/MCB.00675-09 19822665 PMC2786867

[B35] Diangelo J. R. Bland M. L. Bambina S. Cherry S. Birnbaum M. J. (2009). The immune response attenuates growth and nutrient storage in *Drosophila* by reducing insulin signaling. Proc. Natl. Acad. Sci. U.S.A. 106, 20853–20858 10.1073/pnas.0906749106 19861550 PMC2791644

[B36] Dillin A. Crawford D. K. Kenyon C. (2002). Timing requirements for insulin/IGF-1 signaling in *C. elegans*. Science 298, 830–834 10.1126/science.1074240 12399591

[B37] Du K. Herzig S. Kulkarni R. N. Montminy M. (2003). TRB3: a tribbles homolog that inhibits Akt/PKB activation by insulin in liver. Science 300, 1574–1577 10.1126/science.1079817 12791994

[B38] Dupont J. Holzenberger M. (2003). Biology of insulin-like growth factors in development. Birth Defects Res. C. Embryo Today 69, 257–271 10.1002/bdrc.10022 14745968

[B39] Elchebly M. Payette P. Michaliszyn E. Cromlish W. Collins S. Loy A. L. (1999). Increased insulin sensitivity and obesity resistance in mice lacking the protein tyrosine phosphatase-1B gene. Science 283, 1544–1548 10.1126/science.283.5407.1544 10066179

[B40] Emerson K. J. Bradshaw W. E. Holzapfel C. M. (2009). Complications of complexity: integrating environmental, genetic and hormonal control of insect diapause. Trends Genet. 25, 217–225 10.1016/j.tig.2009.03.009 19375812

[B41] Fayard E. Tintignac L. A. Baudry A. Hemmings B. A. (2005). Protein kinase B/Akt at a glance. J. Cell. Sci. 118, 5675–5678 10.1242/jcs.02724 16339964

[B42] Felix T. M. Hughes K. A. Stone E. A. Drnevich J. M. Leips J. (2012). Age-specific variation in immune response in *Drosophila melanogaster* has a genetic basis. Genetics 191, 989–1002 10.1534/genetics.112.140640 22554890 PMC3389989

[B43] Fernandez R. Tabarini D. Azpiazu N. Frasch M. Schlessinger J. (1995). The *Drosophila* insulin receptor homolog: a gene essential for embryonic development encodes two receptor isoforms with different signaling potential. EMBO J. 14, 3373–3384 7628438 10.1002/j.1460-2075.1995.tb07343.x PMC394404

[B45] Flatt T. Heyland A. Rus F. Porpiglia E. Sherlock C. Yamamoto R. (2008a). Hormonal regulation of the humoral innate immune response in *Drosophila melanogaster*. J. Exp. Biol. 211, 2712–2724 10.1242/jeb.014878 18689425 PMC2522372

[B46] Flatt T. Min K. J. D'alterio C. Villa-Cuesta E. Cumbers J. Lehmann R. (2008b). *Drosophila* germ-line modulation of insulin signaling and lifespan. Proc. Natl. Acad. Sci. U.S.A. 105, 6368–6373 18434551 10.1073/pnas.0709128105 PMC2359818

[B44] Flatt T. Kawecki T. J. (2007). Juvenile hormone as a regulator of the trade-off between reproduction and life span in *Drosophila melanogaster*. Evolution 61, 1980–1991 10.1111/j.1558-5646.2007.00151.x 17683439

[B47] Flatt T. Tu M. P. Tatar M. (2005). Hormonal pleiotropy and the juvenile hormone regulation of *Drosophila* development and life history. Bioessays 27, 999–1010 10.1002/bies.20290 16163709

[B48] Frame S. Cohen P. Biondi R. M. (2001). A common phosphate binding site explains the unique substrate specificity of GSK3 and its inactivation by phosphorylation. Mol. Cell 7, 1321–1327 10.1016/S1097-2765(01)00253-2 11430833

[B49] Garsin D. A. Villanueva J. M. Begun J. Kim D. H. Sifri C. D. Calderwood S. B. (2003). Long-lived *C. elegans* daf-2 mutants are resistant to bacterial pathogens. Science 300, 1921 10.1126/science.1080147 12817143

[B50] Gershman B. Puig O. Hang L. Peitzsch R. M. Tatar M. Garofalo R. S. (2007). High-resolution dynamics of the transcriptional response to nutrition in *Drosophila*: a key role for dFOXO. Physiol. Genomics 29, 24–34 10.1152/physiolgenomics.00061.2006 17090700

[B51] Giannakou M. E. Partridge L. (2007). Role of insulin-like signalling in *Drosophila* lifespan. Trends Biochem. Sci. 32, 180–188 10.1016/j.tibs.2007.02.007 17412594

[B52] Giannakou M. E. Goss M. Junger M. A. Hafen E. Leevers S. J. Partridge L. (2004). Long-lived *Drosophila* with overexpressed dFOXO in adult fat body. Science 305, 361 10.1126/science.1098219 15192154

[B53] Goberdhan D. C. Paricio N. Goodman E. C. Mlodzik M. Wilson C. (1999). *Drosophila* tumor suppressor PTEN controls cell size and number by antagonizing the Chico/PI3-kinase signaling pathway. Genes Dev. 13, 3244–3258 10.1101/gad.13.24.3244 10617573 PMC317204

[B54] Golden J. W. Riddle D. L. (1982). A pheromone influences larval development in the nematode *Caenorhabditis elegans*. Science 218, 578–580 10.1126/science.6896933 6896933

[B55] Golden J. W. Riddle D. L. (1984). The *Caenorhabditis elegans* dauer larva: developmental effects of pheromone, food, and temperature. Dev. Biol. 102, 368–378 10.1016/0012-1606(84)90201-X 6706004

[B56] Goto S. G. (2013). Roles of circadian clock genes in insect photoperiodism. Entomol. Sci. 16, 1–16 10.1111/ens.12000

[B57] Goto S. G. Shiga S. Numata H. (2010). Photoperiodism in insects: perception of light and the role of clock genes, in Photoperiodism, eds. Nelson R J. Denlinger D. L. Somers D. E. (Oxford: Oxford University Press) 258–286

[B58] Gottlieb S. Ruvkun G. (1994). daf-2, daf-16 and daf-23: genetically interacting genes controlling Dauer formation in *Caenorhabditis elegans*. Genetics 137, 107–120 8056303 10.1093/genetics/137.1.107 PMC1205929

[B59] Gracey A. Y. Fraser E. J. Li W. Fang Y. Taylor R. R. Rogers J. (2004). Coping with cold: an integrative, multitissue analysis of the transcriptome of a poikilothermic vertebrate. Proc. Natl. Acad. Sci. U.S.A. 101, 16970–16975 10.1073/pnas.0403627101 15550548 PMC534716

[B60] Gronke S. Clarke D. F. Broughton S. Andrews T. D. Partridge L. (2010). Molecular evolution and functional characterization of *Drosophila* insulin-like peptides. PLoS Genet. 6:e1000857 10.1371/journal.pgen.1000857 20195512 PMC2829060

[B61] Hahn D. A. Denlinger D. L. (2007). Meeting the energetic demands of insect diapause: nutrient storage and utilization. J. Insect Physiol. 53, 760–773 10.1016/j.jinsphys.2007.03.018 17532002

[B62] Hahn D. A. Denlinger D. L. (2011). Energetics of diapause. Annu. Rev. Entomol. 56, 103–121 10.1146/annurev-ento-112408-085436 20690828

[B63] Hamilton B. Dong Y. Shindo M. Liu W. Odell I. Ruvkun G. (2005). A systematic RNAi screen for longevity genes in *C*. elegans. Genes Dev. 19, 1544–1555 10.1101/gad.1308205 15998808 PMC1172061

[B64] Hanada M. Feng J. Hemmings B. A. (2004). Structure, regulation and function of PKB/AKT–a major therapeutic target. Biochim. Biophys. Acta 1697, 3–16 10.1016/j.bbapap.2003.11.009 15023346

[B65] Hand S. C. Menze M. A. Borcar A. Patil Y. Covi J. A. Reynolds J. A. (2011). Metabolic restructuring during energy-limited states: insights from *Artemia franciscana* embryos and other animals. J. Insect Physiol. 57, 584–594 10.1016/j.jinsphys.2011.02.010 21335009 PMC3104064

[B66] Hardie J. Baker F. C. Jamieson G. C. Lees A. D. Schooley D. A. (1985). The Identification of an aphid juvenile hormone, and its titer in relation to photoperiod. Physiol. Ent. 10, 297–302 10.1111/j.1365-3032.1985.tb00050.x

[B67] Harrington L. S. Findlay G. M. Gray A. Tolkacheva T. Wigfield S. Rebholz H. (2004). The TSC1-2 tumor suppressor controls insulin-PI3K signaling via regulation of IRS proteins. J. Cell Biol. 166, 213–223 10.1083/jcb.200403069 15249583 PMC2172316

[B68] Harris T. E. Lawrence J. C. Jr. (2003). TOR signaling. Sci. STKE 2003:re15 14668532 10.1126/stke.2122003re15

[B69] Herman W. S. (1981). Studies on the adult reproductive diapause of the monarch butterfly, *Danaus plexippus*. Biol. Bull. 160, 89–106 10.2307/1540903

[B70] Herman W. S. Tatar M. (2001). Juvenile hormone regulation of longevity in the migratory monarch butterfly. Proc. Biol. Sci. 268, 2509–2514 10.1098/rspb.2001.1765 11749703 PMC1088908

[B71] Honda Y. Honda S. (1999). The daf-2 gene network for longevity regulates oxidative stress resistance and Mn-superoxide dismutase gene expression in *Caenorhabditis elegans*. FASEB J. 13, 1385–1393 10428762

[B72] Hsu H. J. LaFever L. Drummond-Barbosa D. (2008). Diet controls normal and tumorous germline stem cells via insulin-dependent and -independent mechanisms in *Drosophila*. Dev. Biol. 313, 700–712 10.1016/j.ydbio.2007.11.006 18068153 PMC2254938

[B73] Hwangbo D. S. Gershman B. Tu M. P. Palmer M. Tatar M. (2004). *Drosophila* dFOXO controls lifespan and regulates insulin signalling in brain and fat body. Nature 429, 562–566 10.1038/nature02549 15175753

[B74] Ikeno T. Tanaka S. I. Numata H. Goto S. G. (2010). Photoperiodic diapause under the control of circadian clock genes in an insect. BMC Biol. 8:116 10.1186/1741-7007-8-116 20815865 PMC2942818

[B75] Ikeya T. Galic M. Belawat P. Nairz K. Hafen E. (2002). Nutrient-dependent expression of insulin-like peptides from neuroendocrine cells in the CNS contributes to growth regulation in *Drosophila*. Curr. Biol. 12, 1293–1300 10.1016/S0960-9822(02)01043-6 12176357

[B76] Ito C. Goto S. G. Shiga S. Tomioka K. Numata H. (2008). Peripheral circadian clock for the cuticle deposition rhythm in *Drosophila melanogaster*. Proc. Natl. Acad. Sci. U.S.A. 105, 8446–8451 10.1073/pnas.0800145105 18539772 PMC2448856

[B77] Junger M. A. Rintelen F. Stocker H. Wasserman J. D. Vegh M. Radimerski T. (2003). The *Drosophila* forkhead transcription factor FOXO mediates the reduction in cell number associated with reduced insulin signaling. J. Biol. 2, 20 10.1186/1475-4924-2-20 12908874 PMC333403

[B78] Kapahi P. Zid B. M. Harper T. Koslover D. Sapin V. Benzer S. (2004). Regulation of lifespan in *Drosophila* by modulation of genes in the TOR signaling pathway. Curr. Biol. 14, 885–890 10.1016/j.cub.2004.03.059 15186745 PMC2754830

[B79] Kapan N. Lushchak O. V. Luo J. Nassel D. R. (2012). Identified peptidergic neurons in the *Drosophila* brain regulate insulin-producing cells, stress responses and metabolism by coexpressed short neuropeptide F and corazonin. Cell Mol Life Sci. 69, 4051–4066 10.1007/s00018-012-1097-z 22828865 PMC11114645

[B80] Khani A. Moharramipour S. (2010). Cold hardiness and supercooling capacity in the overwintering larvae of the codling moth, *Cydia pomonella*. J. Insect Sci. 10, 83 10.1673/031.010.8301 20673068 PMC3383407

[B81] Kimura K. D. Tissenbaum H. A. Liu Y. Ruvkun G. (1997). daf-2, an insulin receptor-like gene that regulates longevity and diapause in *Caenorhabditis elegans*. Science 277, 942–946 10.1126/science.277.5328.942 9252323

[B82] Kitisin K. Saha T. Blake T. Golestaneh N. Deng M. Kim C. (2007). Tgf-Beta signaling in development. Sci. STKE 2007:cm1 10.1126/stke.3992007cm1 17699101

[B83] Kops G. J. Dansen T. B. Polderman P. E. Saarloos I. Wirtz K. W. Coffer P. J. (2002a). Forkhead transcription factor FOXO3a protects quiescent cells from oxidative stress. Nature 419, 316–321 10.1038/nature01036 12239572

[B84] Kops G. J. Medema R. H. Glassford J. Essers M. A. Dijkers P. F. Coffer P. J. (2002b). Control of cell cycle exit and entry by protein kinase B-regulated forkhead transcription factors. Mol. Cell. Biol. 22, 2025–2036 10.1128/MCB.22.7.2025-2036.2002 11884591 PMC133681

[B85] Kostal V. (2011). Insect photoperiodic calendar and circadian clock: independence, cooperation, or unity. J. Insect Physiol. 57, 538–556 10.1016/j.jinsphys.2010.10.006 21029738

[B86] Kostal V. Denlinger D. L. (2011). Dormancy and developmental arrest in invertebrates. J. Insect Physiol. 57, 537 10.1016/j.jinsphys.2011.04.001 21501619

[B87] Kramer J. M. Davidge J. T. Lockyer J. M. Staveley B. E. (2003). Expression of *Drosophila* FOXO regulates growth and can phenocopy starvation. BMC Dev. Biol. 3:5 10.1186/1471-213X-3-5 12844367 PMC183841

[B89] Lee K. S. Kwon O. Y. Lee J. H. Kwon K. Min K. J. Jung S. A. (2008). *Drosophila* short neuropeptide F signalling regulates growth by ERK-mediated insulin signalling. Nat. Cell Biol. 10, 468–475 10.1038/ncb1710 18344986

[B90] Leevers S. J. (2001). Growth control: invertebrate insulin surprises! Curr. Biol. 11, R209–R212 10.1016/S0960-9822(01)00107-5 11301264

[B91] LeFever L. Drummond-Barbosa D. (2005). Direct control of germline stem cell division and cyst growth by neural insulin in *Drosophila*. Science 309, 1071–1073 10.1126/science.1111410 16099985

[B92] Libina N. Berman J. R. Kenyon C. (2003). Tissue-specific activities of *C. elegans* DAF-16 in the regulation of lifespan. Cell 115, 489–502 10.1016/S0092-8674(03)00889-4 14622602

[B93] Logan C. Y. Nusse R. (2004). The Wnt signaling pathway in development and disease. Annu. Rev. Cell Dev. Biol. 20, 781–810 10.1146/annurev.cellbio.20.010403.113126 15473860

[B94] Luckhart S. Riehle M. A. (2007). The insulin signaling cascade from nematodes to mammals: insights into innate immunity of *Anopheles* mosquitoes to malaria parasite infection. Dev. Comp. Immunol. 31, 647–656 10.1016/j.dci.2006.10.005 17161866 PMC2233911

[B95] Macrae T. H. (2010). Gene expression, metabolic regulation and stress tolerance during diapause. Cell. Mol. Life Sci. 67, 2405–2424 10.1007/s00018-010-0311-0 20213274 PMC11115916

[B96] Masaki S. (1980). Summer diapause. Annu. Rev. Entomol. 25, 1–25 10.1146/annurev.en.25.010180.000245

[B97] McDonald M. J. Rosbash M. Emery P. (2001). Wild-type circadian rhythmicity is dependent on closely spaced E boxes in the *Drosophila* timeless promoter. Mol. Cell. Biol. 21, 1207–1217 10.1128/MCB.21.4.1207-1217.2001 11158307 PMC99574

[B98] McElwee J. J. Schuster E. Blanc E. Thornton J. Gems D. (2006). Diapause-associated metabolic traits reiterated in long-lived daf-2 mutants in the nematode *Caenorhabditis elegans*. Mech. Ageing Dev. 127, 458–472 10.1016/j.mad.2006.01.006 16522328

[B99] Meelkop E. Temmerman L. Schoofs L. Janssen T. (2011). Signalling through pigment dispersing hormone-like peptides in invertebrates. Prog. Neurobiol. 93, 125–147 10.1016/j.pneurobio.2010.10.004 21040756

[B100] Meuti M. E. Denlinger D. L. (2013). Evolutionary links between circadian clocks and photoperiodic diapause in insects. Integ. Comp. Biol. 53, 131–143 10.1093/icb/ict023 23615363 PMC3687133

[B101] Michaud M. R. Denlinger D. L. (2006). Oleic acid is elevated in cell membranes during rapid cold-hardening and pupal diapause in the flesh fly, *Sarcophaga crassipalpis*. J. Insect Physiol. 52, 1073–1082 10.1016/j.jinsphys.2006.07.005 16997319

[B102] Michaud M. R. Denlinger D. L. (2007). Shifts in the carbohydrate, polyol, and amino acid pools during rapid cold-hardening and diapause-associated cold-hardening in flesh flies (*Sarcophaga crassipalpis*): a metabolomic comparison. J. Comp. Physiol. B 177, 753–763 10.1007/s00360-007-0172-5 17576567

[B103] Miller B. S. Shankavaram U. T. Horney M. J. Gore A. C. Kurtz D. T. Rosenzweig S. A. (1996). Activation of cJun NH2-terminal kinase/stress-activated protein kinase by insulin. Biochemistry 35, 8769–8775 10.1021/bi952651r 8679641

[B104] Murphy C. T. Mccarroll S. A. Bargmann C. I. Fraser A. Kamath R. S. Ahringer J. (2003). Genes that act downstream of DAF-16 to influence the lifespan of *Caenorhabditis elegans*. Nature 424, 277–283 10.1038/nature01789 12845331

[B105] Murray P. Hayward S. A. Govan G. G. Gracey A. Y. Cossins A. R. (2007). An explicit test of the phospholipid saturation hypothesis of acquired cold tolerance in *Caenorhabditis elegans*. Proc. Natl. Acad. Sci. U.S.A. 104, 5489–5494 10.1073/pnas.0609590104 17369360 PMC1838478

[B106] Numata H. Udaka H. (2010). Photoperiodism in Mollusks in Photoperiodism, eds Nelson R. J. Denlinger D. L. Somers D. E. (Oxford: Oxford University Press), 173–192

[B107] Oh S. W. Mukhopadhyay A. Dixit B. L. Raha T. Green M. R. Tissenbaum H. A. (2006). Identification of direct DAF-16 targets controlling longevity, metabolism and diapause by chromatin immunoprecipitation. Nat. Genet. 38, 251–257 10.1038/ng1723 16380712

[B108] Partridge L. Piper M. D. Mair W. (2005). Dietary restriction in *Drosophila*. Mech. Ageing Dev. 126, 938–950 10.1016/j.mad.2005.03.023 15935441

[B109] Poelchau M. F. Reynolds J. A. Denlinger D. L. Elsik C. G. Armbruster P. A. (2011). A de novo transcriptome of the Asian tiger mosquito, *Aedes albopictus*, to identify candidate transcripts for diapause preparation. BMC Genomics 12:619 10.1186/1471-2164-12-619 22185595 PMC3258294

[B110] Puig O. Marr M. T. Ruhf M. L. Tjian R. (2003). Control of cell number by *Drosophila* FOXO: downstream and feedback regulation of the insulin receptor pathway. Genes Dev. 17, 2006–2020 10.1101/gad.1098703 12893776 PMC196255

[B111] Raff R. (1996). The Shape of Life: Genes, Development, and the Evolution of Animal Form. Chicago, IL: University Chicago Press

[B112] Ragland G. J. Denlinger D. L. Hahn D. A. (2010). Mechanisms of suspended animation are revealed by transcript profiling of diapause in the flesh fly. Proc. Natl. Acad. Sci. U.S.A. 107, 14909–14914 10.1073/pnas.1007075107 20668242 PMC2930464

[B113] Ragland G. J. Egan S. P. Feder J. L. Berlocher S. H. Hahn D. A. (2011). Developmental trajectories of gene expression reveal candidates for diapause termination: a key life-history transition in the apple maggot fly *Rhagoletis pomonella*. J. Exp. Biol. 214, 3948–3959 10.1242/jeb.061085 22071185

[B114] Reynolds J. A. Poelchau M. F. Rahman Z. Armbruster P. A. Denlinger D. L. (2012). Transcript profiling reveals mechanisms for lipid conservation during diapause in the mosquito, *Aedes albopictus*. J. Insect Physiol. 58, 966–973 10.1016/j.jinsphys.2012.04.013 22579567 PMC3389261

[B115] Robich R. M. Denlinger D. L. (2005). Diapause in the mosquito *Culex pipiens* evokes a metabolic switch from blood feeding to sugar gluttony. Proc. Natl. Acad. Sci. U.S.A. 102, 15912–15917 10.1073/pnas.0507958102 16247003 PMC1276097

[B116] Rulifson E. J. Kim S. K. Nusse R. (2002). Ablation of insulin-producing neurons in flies: growth and diabetic phenotypes. Science 296, 1118–1120 10.1126/science.1070058 12004130

[B117] Sano H. Kane S. Sano E. Miinea C. P. Asara J. M. Lane W. S. (2003). Insulin-stimulated phosphorylation of a Rab GTPase-activating protein regulates GLUT4 translocation. J. Biol. Chem. 278, 14599–14602 10.1074/jbc.C300063200 12637568

[B118] Saunders D. S. (2010). Photoperiodism in insects: migration and diapause responses in Photoperiodism, eds Nelson R. J. Denlinger D. L. Somers D. E. (Oxford: Oxford University Press), 218–257

[B119] Saunders D. S. (2012). Insect photoperiodism: seeing the light. Physiol. Entomol. 37, 207–218 10.1111/j.1365-3032.2012.00837.x

[B120] Savory F. R. Sait S. M. Hope I. A. (2011). DAF-16 and Delta9 desaturase genes promote cold tolerance in long-lived *Caenorhabditis elegans* age-1 mutants. PLoS ONE 6:e24550 10.1371/journal.pone.0024550 21931751 PMC3169625

[B121] Schmidt P. S. Matzkin L. Ippolito M. Eanes W. F. (2005). Geographic variation in diapause incidence, life-history traits, and climatic adaptation in *Drosophila melanogaster*. Evolution 59, 1721–1732 16331839

[B122] Shiga S. Numata H. (2000). The role of neurosecretory neurons in the *pars intercerebralis* and *pars lateralis* in reproductive diapause of the blowfly, *Protophormia terraenovae*. Naturwissenschaften 87, 125–128 10.1007/s001140050689 10798197

[B123] Shimokawa K. Numata H. Shiga S. (2008). Neurons important for the photoperiodic control of diapause in the bean bug, *Riptortus pedestris*. J. Comp. Physiol. A 194, 751–762 10.1007/s00359-008-0346-y 18546002

[B124] Shmookler Reis R. J. Xu L. Lee H. Chae M. Thaden J. J. Bharill P. (2011). Modulation of lipid biosynthesis contributes to stress resistance and longevity of *C. elegans* mutants. Aging (Albany NY) 3, 125–147 21386131 10.18632/aging.100275 PMC3082008

[B125] Sim C. Denlinger D. L. (2008). Insulin signaling and FOXO regulate the overwintering diapause of the mosquito *Culex pipiens*. Proc. Natl. Acad. Sci. U.S.A. 105, 6777–6781 10.1073/pnas.0802067105 18448677 PMC2373331

[B126] Sim C. Denlinger D. L. (2009a). A shut-down in expression of an insulin-like peptide, ILP-1, halts ovarian maturation during the overwintering diapause of the mosquito *Culex pipiens*. Insect Mol. Biol. 18, 325–332 10.1111/j.1365-2583.2009.00872.x 19523064 PMC3835429

[B127] Sim C. Denlinger D. L. (2009b). Transcription profiling and regulation of fat metabolism genes in diapausing adults of the mosquito *Culex pipiens*. Physiol. Genomics 39, 202–209 10.1152/physiolgenomics.00095.2009 19706691 PMC2789672

[B128] Sim C. Denlinger D. L. (2011). Catalase and superoxide dismutase-2 enhance survival and protect ovaries during overwintering diapause in the mosquito *Culex pipiens*. J. Insect Physiol. 57, 628–634 10.1016/j.jinsphys.2011.01.012 21277308 PMC3104096

[B129] Sim C. Denlinger D. L. (2013). Juvenile hormone III suppresses forkhead of transcription factor in the fat body and reduces fat accumulation in the diapausing mosquito, *Culex pipiens*. Insect Mol. Biol. 22, 1–11 10.1111/j.1365-2583.2012.01166.x 23121109

[B130] Soderberg J. A. Carlsson M. A. Nassel D. R. (2012). Insulin-producing cells in the *Drosophila* brain also express satiety-inducing cholecystokinin-like peptide, drosulfakinin. Front. Endocrinol. (Lausanne) 3:109 10.3389/fendo.2012.00109 22969751 PMC3431609

[B131] Sousa-Nunes R. Yee L. L. Gould A. P. (2011). Fat cells reactivate quiescent neuroblasts via TOR and glial insulin relays in *Drosophila*. Nature 471, 508–512 10.1038/nature09867 21346761 PMC3146047

[B132] Sun J. Folk D. Bradley T. J. Tower J. (2002). Induced overexpression of mitochondrial Mn-superoxide dismutase extends the life span of adult *Drosophila melanogaster*. Genetics 161, 661–672 12072463 10.1093/genetics/161.2.661 PMC1462135

[B133] Tammariello S. P. Denlinger D. L. (1998). G_0_/G_1_ cell cycle arrest in the brain of *Sarcophaga crassipalpis* during pupal diapause and the expression pattern of the cell cycle regulator, Proliferating Cell Nuclear Antigen. Insect Biochem. Mol. Biol. 28, 83–89 10.1016/S0965-1748(97)00082-9 9639874

[B134] Taniguchi C. M. Emanuelli B. Kahn C. R. (2006). Critical nodes in signalling pathways: insights into insulin action. Nat. Rev. Mol. Cell Biol. 7, 85–96 10.1038/nrm1837 16493415

[B135] Tatar M. Kopelman A. Epstein D. Tu M. P. Yin C. M. Garofalo R. S. (2001). A mutant *Drosophila* insulin receptor homolog that extends life-span and impairs neuroendocrine function. Science 292, 107–110 10.1126/science.1057987 11292875

[B136] Tatar M. Yin C. (2001). Slow aging during insect reproductive diapause: why butterflies, grasshoppers and flies are like worms. Exp. Gerontol. 36, 723–738 10.1016/S0531-5565(00)00238-2 11295511

[B137] Tauber M. J. Tauber C. A. Masaki S. (1986). Seasonal Adaptations of Insects. New York, NY: Oxford University Press

[B138] Tu M. P. Yin C. M. Tatar M. (2005). Mutations in insulin signaling pathway alter juvenile hormone synthesis in *Drosophila melanogaster*. Gen. Comp. Endocrinol. 142, 347–356 10.1016/j.ygcen.2005.02.009 15935161

[B139] Ueki K. Kondo T. Kahn C. R. (2004). Suppressor of cytokine signaling 1 (SOCS-1) and SOCS-3 cause insulin resistance through inhibition of tyrosine phosphorylation of insulin receptor substrate proteins by discrete mechanisms. Mol. Cell. Biol. 24, 5434–5446 10.1128/MCB.24.12.5434-5446.2004 15169905 PMC419873

[B140] Vafopoulou X. Cardinal-Aucoin M. Steel C. G. (2012). Rhythmic release of prothoracicotropic hormone from the brain of an adult insect during egg development. Comp. Biochem. Physiol. A Mol. Integr. Physiol. 161, 193–200 10.1016/j.cbpa.2011.10.026 22079105

[B141] Vafopoulou X. Steel C. G. Terry K. L. (2007). Neuroanatomical relations of prothoracicotropic hormone neurons with the circadian timekeeping system in the brain of larval and adult *Rhodnius prolixus* (Hemiptera). J. Comp. Neurol. 503, 511–524 10.1002/cne.21393 17534946

[B142] Vanfleteren J. R. De Vreese A. (1995). The gerontogenes age-1 and daf-2 determine metabolic rate potential in aging *Caenorhabditis elegans*. FASEB J. 9, 1355–1361 7557026 10.1096/fasebj.9.13.7557026

[B143] Vereshchagina N. Ramel M. C. Bitoun E. Wilson C. (2008). The protein phosphatase PP2A-B' subunit Widerborst is a negative regulator of cytoplasmic activated Akt and lipid metabolism in *Drosophila*. J. Cell. Sci. 121, 3383–3392 10.1242/jcs.035220 18827008

[B144] Vesala L. Hoikkala A. (2011). Effects of photoperiodically induced reproductive diapause and cold hardening on the cold tolerance of *Drosophila montana*. J. Insect Physiol. 57, 46–51 10.1016/j.jinsphys.2010.09.007 20932841

[B145] Walker G. A. Lithgow G. J. (2003). Lifespan extension in *C. elegans* by a molecular chaperone dependent upon insulin-like signals. Aging Cell 2, 131–139 10.1046/j.1474-9728.2003.00045.x 12882326

[B146] Wang J. Kim S. K. (2003). Global analysis of dauer gene expression in *Caenorhabditis elegans*. Development 130, 1621–1634 10.1242/dev.00363 12620986

[B147] Wang M. C. Bohmann D. Jasper H. (2005). JNK extends life span and limits growth by antagonizing cellular and organism-wide responses to insulin signaling. Cell 121, 115–125 10.1016/j.cell.2005.02.030 15820683

[B148] Watanabe H. Pan Z. Q. Schreiber-Agus N. Depinho R. A. Hurwitz J. Xiong Y. (1998). Suppression of cell transformation by the cyclin-dependent kinase inhibitor p57KIP2 requires binding to proliferating cell nuclear antigen. Proc. Natl. Acad. Sci. U.S.A. 95, 1392–1397 10.1073/pnas.95.4.1392 9465025 PMC19016

[B149] Williams K. D. Busto M. Suster M. L. So A. K. Ben-Shahar Y. Leevers S. J. (2006). Natural variation in *Drosophila melanogaster* diapause due to the insulin-regulated PI3-kinase. Proc. Natl. Acad. Sci. U.S.A. 103, 15911–15915 10.1073/pnas.0604592103 17043223 PMC1635102

[B150] Williams K. D. Schmidt P. S. Sokolowski M. B. (2010). Photoperiodism in insects: molecular basis and consequences of diapause in Photoperiodism, eds Nelson R. J. Denlinger D. L. Somers D. E. (Oxford: Oxford University Press), 287–317

[B151] Wittwer F. Jaquenoud M. Brogiolo W. Zarske M. Wustemann P. Fernandez R. (2005). Susi, a negative regulator of *Drosophila* PI3-kinase. Dev. Cell 8, 817–827 10.1016/j.devcel.2005.04.002 15935772

[B152] Xu W. H. Lu Y. X. Denlinger D. L. (2012). Cross-talk between the fat body and brain regulates insect developmental arrest. Proc. Natl. Acad. Sci. U.S.A. 109, 14687–14692 10.1073/pnas.1212879109 22912402 PMC3437847

[B153] Zhang H. Liu J. Li C. R. Momen B. Kohanski R. A. Pick L. (2009). Deletion of *Drosophila* insulin-like peptides causes growth defects and metabolic abnormalities. Proc. Natl. Acad. Sci. U.S.A. 106, 19617–19622 10.1073/pnas.0905083106 19887630 PMC2780814

[B154] Zheng X. Sehgal A. (2010). AKT and TOR signaling set the pace of the circadian pacemaker. Curr. Biol. 20, 1203–1208 10.1016/j.cub.2010.05.027 20619819 PMC3165196

